# Investigating the toll of childhood socioeconomic disadvantage on adolescent mental health in three UK cohorts

**DOI:** 10.1002/jcv2.70131

**Published:** 2026-07-11

**Authors:** Caitlyn Rawers, Orla McBride, Jamie Murphy, Eoin McElroy

**Affiliations:** ^1^ School of Psychology Ulster University Coleraine UK

**Keywords:** ALSPAC, cross‐cohort comparison, harmonisation, latent class analysis, socioeconomic position, time trends

## Abstract

**Background:**

Low socioeconomic position (SEP) in childhood is robustly associated with adolescent mental health difficulties, yet this relationship is changing over time due to contextual and conceptual differences in these constructs. Consistent SEP and mental health data from different cohorts are required to understand changes over time, which is uncommon. This study used retrospectively harmonised data from three UK birth cohorts to investigate the changing relationship between SEP and mental health between the 1970s and 2000s.

**Methods:**

Mental health and SEP data from three UK birth cohorts (British Cohort Study [BCS70], Avon Longitudinal Study of Parents and Children [ALSPAC], Millennium Cohort Study [MCS]) were harmonised and tested for measurement invariance. Latent factors reflecting internalising, behavioural, and ADHD symptoms, were examined as distal outcomes of SEP latent classes.

**Results:**

Partial scalar invariance was supported with multi‐group confirmatory factor analysis. Although the structure of the SEP latent classes differed considerably across cohorts, the most disadvantaged class in each cohort experienced greater levels of mental health difficulties. In the ALSPAC, only behavioural symptoms were significantly predicted by disadvantaged class membership; in both the BCS70 and MCS, all symptom domains were significant. The strongest association between the most disadvantaged class and mental health symptoms was observed in the MCS.

**Conclusion:**

This study assessed the relationship between SEP and mental health using a novel methodological approach to harmonise and compare cohort data. Findings indicate that mental health may be more strongly related to socioeconomic disadvantage in recent cohorts, despite differences in the structure of SEP. Future research should utilise harmonisation to understand time trends in prevalent societal issues.

## Key Points

**What's known?**

- Robust evidence suggests that socioeconomic disadvantage in early life is associated with poorer mental health in adolescence, although the strength of these relationships may change over time.

**What's new?**

- Results suggest that low socioeconomic position may be more strongly related to poor mental health outcomes, particularly internalising difficulties, in a cohort born in the early 2000s compared to cohorts born in the 1970s and 1990s. Importantly, these differences cannot solely be attributed to the way that mental health scale items were interpreted in each cohort.

**What's relevant?**

- Policymakers need to consider the multidimensionality of socioeconomic position and changes over time to address inequalities in adolescent mental health outcomes.

## INTRODUCTION

Growing up in socioeconomic disadvantage contributes to the development of several prevalent societal issues including mental health difficulties, antisocial behaviour, and substance use problems (Lemstra et al., 2008; Piotrowska et al., 2015; Reiss, 2013). Of these, adolescent mental health issues are highly prevalent, with 15.3% of young people in the UK having at least one mental disorder (Clarke et al., 2020). Socioeconomic inequalities in mental health have been widely studied, and robust evidence suggests that low socioeconomic position in childhood is associated with increased internalising and externalising difficulties in childhood and adolescence (McLaughlin et al., 2011; Peverill et al., 2021; Reiss, 2013). Socioeconomic position (SEP) is a complex and multi‐dimensional construct comprising several areas, including financial, social, and material capital (Krieger et al., 1997). Indeed, there are many different indicators of SEP such as income, education, or housing tenure; however, these are theorised to affect adolescent mental health in different ways. It is posited that socioeconomic disadvantage will primarily affect adolescent mental health and behaviour through stress‐related processes, which increase the likelihood of mental health problems (Sederer et al., 2016; Yoshikawa et al., 2012). Mechanisms are theorised to vary by specific indicators; for instance, household income may affect adolescent mental health via parental stress and psychopathology (Ravensbergen et al., 2026; Yang et al., 2023), whereas parental education may affect mental health via health literacy and knowledge (Xiang et al., 2024). Importantly, the relationship between SEP and adolescent mental health varies depending on the indicator used and researchers have highlighted that they are not interchangeable (Braveman et al., 2005).

Additionally, studies indicate that the relationship between SEP and mental health is changing over time (Collishaw et al., 2019; Gore Langton et al., 2011; Schoon et al., 2003). These changes may be a result of several processes such as changes in the conceptualization and measurement of SEP (Berkman & Macintrye, 1997; Blanden & Machin, 2017), and mental health (McElroy et al., 2020) from between generations. Changes may also be due to differences in the socio‐cultural context of SEP and mental health support or interventions from one generation to the next. For example, Gore Langton et al. (2011) found that low income and rented housing tenure was significantly more strongly related to UK adolescent's internalising difficulties in the early 2000s compared to the 1970s. In particular, the association between low income and adolescent internalising problems was four times higher for the 2000s cohort compared to the 1970s; however, a small proportion of this difference was due to changes in family composition and income (Gore Langton et al., 2011).

Thusly, establishing how childhood SEP affects adolescent mental health over time, as suggested by extant studies (Collishaw et al., 2019; Schoon et al., 2003), necessitates the measurement of SEP and mental health in a consistent and robust way across generations. By extension, SEP and mental health data from multiple cohorts within the same socio‐cultural context is required. Although SEP and mental health are commonly assessed in population‐level studies, the approaches to measuring these concepts can vary considerably, making it difficult to conduct cross‐cohort studies of this relationship (O’Connor et al., 2022). To overcome these discrepancies in measurement, guidance on retrospective harmonisation have been produced (e.g., Fortier et al., 2017); allowing researchers to make substantive comparisons with previously incomparable data.

Focusing on mental health outcomes, this study is one of a series of studies that examines how SEP in the UK has changed and affected important societal issues including adolescent mental health, antisocial behaviour, and substance use between the 1970s and 2000s. Using data from the 1970s British Cohort Study (BCS70), the 1990s Avon Longitudinal Study of Parents and Children (ALSPAC), and the 2000s Millennium Cohort Study (MCS), and adopting an innovative variable harmonisation methodology, these studies have ensured that each construct under investigation has been measured in a consistent and robust manner in each cohort. Given that SEP was central to all studies, the first study in the series harmonised SEP indicators across cohorts, then modelled SEP variation in each cohort using latent class analysis (LCA; Rawers et al., 2025). The study established a distinct SEP profile for each cohort. Despite variations in the features of latent classes, a class characterised by relative socioeconomic disadvantage was common across generations (the ‘*Prestige‐Resource Disadvantaged*’ class). The identification of different SEP profiles across cohorts is unsurprising given the significant societal and policy changes which occurred between the studies (Cracknell et al., 2023; Szreter, 2021). The reduction in the size of the ‘*Prestige‐Resource Disadvantaged*’ class in the MCS (10.0%) compared to the other two cohorts (15.6% [BCS70] and 20.0% [ALSPAC]) is consistent with research that suggests absolute and relative poverty has reduced in the UK since the 1980s and early 1990s (Brewer et al., 2009; Francis‐Devine, 2024; Joyce & Ziliak, 2019). The characteristics of the *‘Prestige‐Resource Disadvantaged’* class in each cohort differed, yet they reflect patterns observed in other studies. For example, social housing and private renting have become increasingly characteristic of the most disadvantaged since the early 2000s (Bailey, 2020; Francis‐Devine, 2024). This corresponds with the increasing proportion of rented or social housing within the ‘*Prestige‐Resource Disadvantaged*’ class across cohorts; in the MCS, 94% lived in social or rented housing, compared to 80% in the BCS70% and 67% in the ALSPAC. Rawers et al. (2025) also identified that sociodemographic characteristics associated with disadvantage or poverty in prior research were consistently related to the ‘*Prestige‐Resource Disadvantaged*’ class in all three cohorts. Thusly, despite using harmonised SEP indicators, differences in indicator patterns were observed and the ‘*Prestige‐Resource Disadvantaged*’ class reflects what socioeconomic disadvantage may have looked like in these generations.

Subsequently, this study focuses on the associations between SEP and adolescent mental health outcomes using the SEP latent class models identified in Rawers et al. (2025) and harmonising parent‐reported adolescent mental health symptoms in the BCS70, ALSPAC, and MCS. A framework for harmonising mental health items has been described previously (see McElroy et al., 2020). Harmonisation is conducted by identifying specific questions/items from different scales that have similar content (i.e., assess similar symptoms), re‐scaling them to comparable metrics, and then testing for measurement invariance (McElroy et al., 2020). If measurement invariance holds, it suggests that the mental health construct being assessed by the scale has the same meaning to each cohort (Putnick & Bornstein, 2016). Few cross‐cohort comparison studies to date have utilised this approach. One exception is a study by McElroy, Tibber, et al. (2023) who harmonised the Rutter Behaviour Scales (Rutter et al., 1970) and Strengths and Difficulties Questionnaire (R. Goodman, 1997) in adolescent samples from four British birth cohorts. After evaluating measurement invariance, they analysed differences in the relationship between four SEP indicators (parental occupational class, maternal and paternal education, and housing tenure) and parent‐reported adolescent internalising and externalising symptoms using a multiple regression model (McElroy, Tibber, et al., 2023). They found a significant increase in the association between housing tenure and both internalising and externalising behaviour for the cohort born in the early 2000s compared to those born in 1958, 1970 and the early 1990s (McElroy, Tibber, et al., 2023). Importantly, since partial scalar invariance was supported, the increase observed in the 2000s cohort could not solely be attributed to how the items were interpreted in the different samples (McElroy, Tibber, et al., 2023).

However, as explored in Rawers et al. (2025), SEP is a multi‐dimensional construct with complex causal relationships between indicators where different indicators capture a unique aspect of SEP (Braveman et al., 2005). Thusly, people may display various levels of advantage and disadvantage by different SEP indicators (P. Müller, 2017). In McElroy, Tibber, et al. (2023), the authors did not account for the multidimensionality of SEP in their analysis and instead examined SEP indicators individually. Rawers et al. (2025) addressed these issues using LCA to understand the complex patterns of SEP in each cohort.

### Study aims

This study aims to extend the work of McElroy, Tibber, et al. (2023) using the SEP latent classes identified by Rawers et al. (2025) to evaluate how the relationship between SEP and mental health has changed over time. The research questions and hypotheses for this study were pre‐registered with the OSF (see link for information). Consistent with prior research, it is hypothesised that parent‐reported internalising and externalising symptoms will be highest in the ‘*Prestige‐Resource Disadvantaged*’ class in each cohort. Additionally, it is expected that the relationship between SEP latent classes and externalising symptoms will be stronger than internalising symptoms in each cohort, as identified in previous studies (Peverill et al., 2021; Reiss, 2013). Finally, internalising and externalising symptoms are expected to be more strongly related to the ‘*Prestige‐Resource Disadvantaged*’ class in the 2000s cohort, similar to the findings of McElroy, Tibber, et al. (2023).

## METHOD

### Studies included

#### The 1970 British Cohort Study

The BCS70 is a birth cohort which include over 16,000 children who were born in one week of March in 1970 across England, Scotland and Wales (University College London [UCL], 2025). As of the date of writing, 10 data collection sweeps have been conducted from birth up to age 46 years in 2016–2018. During the age 46 sweep, 8581 cohort members (CMs) took part. Data have primarily been collected from parents and CMs via face‐to‐face interview or questionnaires; however, teachers and medical examiners have provided data on the CMs. A variety of data have been collected on the CM's development, health, environment, social circumstances, and lifestyle (Sullivan et al., 2023). See the cohort profile for more information (Sullivan et al., 2023). Ethical approval for the BCS70 is granted by the NHS Research Ethics Committee (Shepherd & Gilbert, 2019a).

#### The Avon Longitudinal Study of Parents and Children

The ALSPAC is a regional cohort comprised of pregnant women residing in Avon, UK with expected dates of delivery between 1st April 1991 and 31st December 1992. The initial number of pregnancies enroled was 14,541, with 13,988 children alive at 1 year of age. Additional phases of recruitment were subsequently conducted, resulting in total sample size of 15,477 pregnancies and 14,901 alive at 1 year of age for analyses on data collected after age seven. The phases of enrolment are described in more detail in the cohort profile paper and its update (Boyd et al., 2013; Fraser et al., 2013; Northstone et al., 2019). 14,203 unique mothers were initially enroled in the study and a total of 14,833 unique women (G0 mothers) enroled in ALSPAC as of September 2021 (Fraser et al., 2013). Additionally, 12,113 G0 partners have been in contact with the study by providing data and/or formally enrolling when this started in 2010 and 3807 G0 partners are currently enroled (Northstone et al., 2019). 68 data collection points were conducted between the CM's birth and 18 years (Boyd et al., 2013; Fraser et al., 2013; Northstone et al., 2019). Ethical approval for the study was obtained from the ALSPAC Ethics and Law Committee and the Local Research Ethics Committees. Informed consent for the use of data collected via questionnaires and clinics was obtained from participants following the recommendations of the ALSPAC Ethics and Law Committee at the time. The study website contains details of all the data that is available through a fully searchable data dictionary and variable search tool: http://www.bristol.ac.uk/alspac/researchers/our‐data/.

#### The Millennium Cohort Study

The MCS is a cohort study of children born between 2000 and 2002 in the England, Scotland, Wales and Northern Ireland (UCL, 2024). Seven data collection sweeps have been conducted between 9 months and 17 years as of the date of writing. Over 18,000 CMs took part in the initial survey, and 10,757 took part in the age 17 sweep. Data have been collected from parents, CMs, and teachers via face‐to‐face or web interviews and questionnaires. Data collection have included information on CMs environment, health, development, and behaviour (Connelly & Platt, 2014; Joshi & Fitzsimons, 2016). See cohort profiles for more information (Connelly & Platt, 2014; Joshi & Fitzsimons, 2016). Ethical approval for each sweep is granted by an NHS Research Ethics Committee (Shepherd & Gilbert, 2019b).

### Measures

Table 1 provides details about the harmonised covariates used for analysis, including the data collection sweep the variables were taken from. See Supporting Information S1: Appendix S1 for a full harmonisation table which includes information about the original variables and recoding. The pre‐registration provides information for the data discoverability and harmonisation of the variables used in the present study.

**TABLE 1:** Harmonised SEP indicator and covariate descriptions—Adapted from Rawers et al. (2025).

	CM age			Description	Responses
	BCS70	ALSPAC	MCS		
SEP indicator					
Social class	10 years	Birth‐8 years	11 years	Highest social class of either parent	0 = non‐manual occupation 1 = manual occupation
Income	10 years	11 years	11 years	Total household income, equivalized by household size	0 = higher income 1 = low income
Housing tenure	10 years	10 years	11 years	Housing tenure of accommodation	0 = Owned or mortgaged 1 = rented or other
Education	5–10 years	Birth‐8 years	11 years	Highest educational or equivalent qualification of either parent	0 = A‐levels or higher 1 = less than A‐levels
Car access	10 years	10 years	11 years	Parents own or have access to at least one car	0 = Yes, access to car 1 = No access to car
Mould/damp in accommodation	10 years	10 years	11 years	Prescence of mould or damp in accommodation	0 = No mould/damp 1 = Yes, mould/damp present
Overcrowding	10 years	10 years	11 years	Ratio of total number of people to number of rooms for living/sleeping	0 = No overcrowding (<2 per room) 1 = Overcrowding (≥2 per room)
Covariate					
CM sex	–	–	9 months‐17 years	CM's sex	0 = male 1 = female
CM ethnicity	5–34 years	Birth	9 months‐14 years	CM's ethnic group classification (6 census categories)	0 = ethnic majority (white) 1 = ethnic minority
Number of siblings	16 years	16 years	17 years	Number of other siblings in household, not including CM	Number of siblings
Marital status	16 years	16 years	17 years	Carers' marital status	0 = Married* 1 = cohabiting 2 = single
CM chronic illness	16 years	16 years	17 years	CM has a longstanding physical/mental illness lasting 12 months or more	0 = No chronic illness 1 = Yes, chronic illness
Parental chronic illness	16 years	Birth‐16 years	14 years	Either parent has a longstanding physical illness lasting 12 months or more	0 = No chronic illness 1 = Yes, chronic illness

*Note*: Items marked with an asterisk (*) were the reference category when variables were dummy coded for analysis.

### SEP latent classes

SEP latent classes were previously derived in Rawers et al. (2025) using seven SEP indicators (refer to Table 1 for variable details). In the BCS70, four latent classes were identified: ‘*Prestige‐Resource Advantaged*’ (40.0%, *n* = 3377), ‘*Prestige Disadvantaged*’ (20.8%, *n* = 1755), ‘*Prestige‐Housing Disadvantaged*’ (23.6%, *n* = 1991), and ‘*Prestige‐Resource Disadvantaged*’ (15.6%, *n* = 1313) classes. In the ALSPAC, only two latent classes were identified: a ‘*Prestige‐Resource Advantaged*’ (80.0%, *n* = 5115) and ‘*Prestige‐Resource Disadvantaged*’ (20.0%, *n* = 1280) class. In the MCS, three latent classes were identified: ‘*Prestige‐Resource Advantaged*” (74.2%, *n* = 6417), ‘*Prestige‐Housing Disadvantaged*’ (15.8%, *n* = 1367), and ‘*Prestige‐Resource Disadvantaged*’ (10.0%, *n* = 864) classes. See Rawers et al. (2025) for more details on latent class identification. See Table S1 for conditional item response probabilities for the latent classes in each cohort.

### Mental health outcomes

In the BCS70, adolescent mental health symptoms were assessed via the Rutter Behaviour Scale (Rutter et al., 1970) at age 16. In the ALSPAC, the Strengths and Difficulties Questionnaire (SDQ; R. Goodman, 1997) was administered at age 16. In the MCS, the SDQ was administered at age 17. For all three cohorts, data from parent‐reported versions of the scales were used to maintain consistency across reporters. Items from the Rutter Behaviour Scale and SDQ differ in wording but overlap in content; previous research indicates that partial scalar invariance was supported for these cohorts in adolescence (McElroy et al., 2020). In this study, comparable items related to internalising, behavioural, and attention/hyperactivity (ADHD) difficulties were identified using the online Harmony software (Wood et al., 2022). Harmony uses Sentence‐BERT (Reimers & Gurevych, 2019), a natural language processing model, to compare the similarity of question items based on their underlying semantic content; paired items are then assigned a cosine value between −1 and 1, where values closer to 1 indicate greater similarity (McElroy, Moltrect, et al., 2023). Following identification by Harmony, items were manually screened to ensure no potential matches were missed. Items on the Rutter Scale were originally scored on a three‐point Likert scale from 1 (‘*Certainly applies*’) to 3 (‘*Doesn't apply*’). Responses to this scale were reverse coded to reflect the three‐point Likert scale used in the SDQ (1 = ‘*Not true*’ to 3 = ‘*Certainly true*’), thusly higher values reflected higher severity of symptoms.

### Analytic sample

CMs were included in the analytic sample if the met the following criteria: they were a singleton or first‐born twin or triplet; they had valid ethnicity and sex data; they had at least one non‐missing SEP indicator; at least one non‐missing mental health outcome variable; and, as this study was part of a series, at least one non‐missing antisocial behaviour and substance use outcome variable. See the pre‐registration here for further details. The final analytical sample for each cohort included: BCS70 (*n* = 8436), ALSPAC (*n* = 6395), and MCS (*n* = 8548).

### Data analysis

Missing data were addressed in the present study using imputation and non‐response weighting. Non‐response weights were derived as the inverse of participant's probability of response at the age 16 sweep of the BCS70, phase 4 of the ALSPAC, and age 17 wave of the MCS. Response probability was estimated using a logistic regression model that included harmonised covariates from the first sweep of each cohort (maternal age at birth, birthweight, number of children in the household, CM ethnicity, and lone parenting). See Rawers et al. (2025) for more details on non‐response weight derivation.

Additionally, partial missing covariate and outcome data were imputed in each cohort using the *missForest* package in R (Rawers et al., 2025; Stekhoven, 2022; see). Out‐of‐bag (OOB) errors were used to assess the imputed covariate and outcome data. Mean square error (MSE) values were computed for continuous variables and the proportion falsely classified (PFC) were computed for categorical variables (Stekhoven, 2011). For both MSE and PFC, values closer to 0 are desirable (Stekhoven, 2011). See Table S2 for OOB errors and details.

First, scale items for internalising, behavioural, and ADHD symptoms were tested for measurement invariance using a multi‐group confirmatory factor analysis (MGCFA) model in Mplus 8.9 with the WLMSV estimator (Muthén & Muthén, 2017). The level of measurement invariance that holds dictates the type of comparisons that can be made across cohorts (McElroy et al., 2020; Putnick & Bornstein, 2016). If the same measurement model structure exists in all cohorts (i.e., the number of factors and loading patterns are the same), *configural invariance* is supported (Putnick & Bornstein, 2016). *Metric invariance* was tested by constraining factor loadings to be equal in each cohort and then fit is compared to the configural model. If metric invariance is supported, the relationship between the latent variable and the items are consistent across cohorts and factor variances and covariances can be compared across groups substantively (Putnick & Bornstein, 2016). For *scalar invariance*, the factor loadings and intercepts for each item are constrained to equal in each cohort. If this is supported, it suggests that participants in each cohort are interpreting the items in a similar manner and factor means can be compared across cohorts (Putnick & Bornstein, 2016). However, researchers have noted that full scalar invariance rarely holds in practise (Van De Schoot et al., 2015). If full scalar invariance is not supported, then partial invariance (i.e., freeing the loading/threshold of specific items) can still allow comparisons to be made (Byrne et al., 1989).

To assess measurement invariance, several fit statistics were evaluated. Although differences in the chi‐square statistic across models are frequently reported, this is a poor method of judging invariance as it is too sensitive to small model misfits in large samples (Chen, 2007). Overall model fit for each model was assessed with the comparative fit index (CFI; Bentler, 1990), Tucker‐Lewis index (TLI; Tucker & Lewis, 1973), root mean square error of approximation (RMSEA; Browne & Cudeck, 1992), and the Standardised Root Mean Square Residual (SRMR; Jöreskog & Sörbom, 1982). These values were evaluated against conventional standards where CFI and TLI values ≥0.90 and RMSEA and SRMR values ≤0.08 reflect adequate model fit (Hu & Bentler, 1999). Nested models were compared based on changes in fit indices, specifically <−0.01 change in CFI and <0.015 change in RMSEA and SRMR (<0.015) values (Chen, 2007).

Once the mental health items were tested for measurement invariance, they were evaluated as a distal outcome in the weighted LCA models established in Rawers et al. (2025). CMs were classified according to their most likely SEP latent class using the CPROBABILITIES command in Mplus (Muthén & Muthén, 2017). In a separate model, SEP latent classes were dummy‐coded with the ‘*Prestige‐Resource Advantaged*’ class as the reference category. Participants' factor scores for internalising, behavioural, and ADHD symptom from the MGCFA model were regressed on SEP latent class membership and demographic covariates. Non‐response weights were included for each cohort (see Rawers et al., 2025). In the MCS, the previously derived cluster and stratum variables were also included to account for the purposeful oversampling of disadvantaged areas and ethnic minorities in this study (Plewis, 2007).

## RESULTS

### Descriptive statistics

Descriptive statistics for sociodemographic covariates in each cohort are presented in Table 2. The mean number of siblings was 1.40 (SD = 1.03) in the BCS70, 1.34 (SD = 0.75) in the ALSPAC, and 1.34 (SD = 1.10) in the MCS.

**TABLE 2:** Descriptive statistics of harmonised covariates.

Covariate	BCS70		ALSPAC		MCS	
	*n*	%	*n*	%	*n*	%
CM sex						
Male	4132	49.0%	2924	45.7%	4289	49.6%
Female	4304	51.0%	3471	54.3%	4359	50.4%
CM ethnicity						
Ethnic majority	8176	96.9%	6143	96.1%	6993	80.9%
Ethnic minority	260	3.1%	252	3.9%	1655	19.1%
Marital status						
Married	7665	90.9%	5093	79.6%	5415	62.6%
Cohabiting	94	1.1%	354	5.5%	869	10.0%
Single	677	8.0%	948	14.8%	2364	27.3%
CM chronic illness						
Yes	1210	14.3%	544	8.5%	1454	16.8%
No	7226	85.7%	5851	91.5%	7194	83.2%
Parental chronic illness						
Yes	2383	28.2%	1219	19.1%	2896	33.5%
No	6053	71.8%	5176	80.9%	5752	66.5%

*Note*: Some percentages may not add up to 100% due to rounding.

### Measurement invariance

Using Harmony (Wood et al., 2022), items from the Rutter scale (Rutter et al., 1970) and SDQ (R. Goodman, 1997) were assessed for semantic overlap. Cosine values (±1) reflect the similarity of matched items where values closer to 1 reflecting a closer match. Some cosine values were low (<0.40) specifically for the ‘*Irritability/temper*’ and ‘*Stealing*’ constructs; however, these items were manually reviewed and included based on researcher judgement. Low cosine values may reflect colloquial language used for these items (e.g., ‘*Quick to fly off the handle*’) that are not easily understood by natural language algorithms. Yet, for majority of item pairs, cosine values reflect high similarity (see Table 3).

**TABLE 3:** Identified item pairs from parent‐reported mental health scales.

Outcome	Construct	Rutter items	SDQ items	Cosine
Internalising	Low mood	Often appears miserable, unhappy or distressed	Often unhappy, down‐hearted and tearful	0.923
	Worry	Often worried, worries about many things	Many worries, often seems worried	0.922
	Fear/anxiety	Tends to be fearful or afraid of new things or new situations	Nervous or clingy in new situations, easily loses confidence	0.618
Behavioural	Irritability/temper	Irritable, quick to fly off the handle	Often has temper tantrums or hot tempers	−0.209
	Dishonesty	Often tells lies	Often lies or cheats	0.877
	Disobedience	Is often disobedient	Generally obedient, usually does what others request^a^	0.553
	Stealing	Sometimes takes things belonging to others	Steals from home, school or elsewhere	0.352
	Aggression	Frequently fights with others	Often fights with other children or bullies them	0.751
ADHD	Restlessness	Very restless, running about or jumping up and down, hardly every still	Restless, overactive, cannot stay still for long	0.708
	Fidgeting	Is squirmy/fidgety	Constantly fidgeting or squirming	0.596
	Concentration problems	Cannot settle to do anything for more than a few moments	Easily distracted, concentration wanders	0.490

^a^ The ‘*Disobedience*’ item in the SDQ was reverse coded for analysis.

Measurement invariance was evaluated via a MGCFA model with three latent factors reflecting internalising, behavioural and ADHD symptoms (see Table 4 for model fit statistics). The configural and metric models demonstrated acceptable model fit and changes in RMSEA, CFI, and TLI values were negligible. However, the CFI for the scalar model reduced by 0.015, more than the recommended value of 0.01 (Chen, 2007). Following the examination of modification indices, the threshold for the ‘*Disobedience*’ item was freed, the CFI value reduced by 0.011, indicating improved fit, despite being marginally outside the recommended range (Chen, 2007). Fit statistics for the partial scalar model indicate that it fit the data well. Thusly, the means for the latent factors may be meaningfully compared across cohorts (Putnick & Bornstein, 2016). Standardised factor loadings of items for each cohort in the configural model are presented in Table 5.

**TABLE 4:** Model fit statistics for measurement invariance of three‐factor multi‐group confirmatory factor analysis (MGCFA) model.

Model	*N*	*χ* ^2^	df	CFI	TLI	RMSEA	SRMR	ΔCFI	ΔTLI	ΔRMSEA	ΔSRMR
Configural	23,479	4450.884	139	0.952	0.943	0.063	0.052	–	–	–	–
Metric	–	4699.299	155	0.950	0.946	0.061	0.055	−0.002	0.003	−0.002	0.003
Scalar	–	5854.962	171	0.937	0.939	0.065	0.070	−0.015	−0.007	0.002	0.018
Partial scalar^a^	–	5437.349	171	0.941	0.944	0.063	0.061	−0.011	−0.002	0.000	0.009

Abbreviations: CFI, comparative fit index; df, degrees of freedom; RMSEA, root mean square error of approximation; SRMR, standardised root mean square residual; TLI, Tucker‐Lewis index.

^a^ Threshold of disobedience item freed.

**TABLE 5:** Standardised factor loadings of three‐factor multi‐group confirmatory factor analysis (MGCFA) Configural model.

Factor	Item	BCS70	ALSPAC	MCS
Internalising	Low mood	0.892	0.899	0.848
	Worry	0.683	0.914	0.773
	Fear/anxiety	0.602	0.808	0.714
Behavioural	Irritability/temper	0.745	0.897	0.789
	Dishonesty	0.825	0.837	0.780
	Disobedience	0.836	0.793	0.559
	Stealing	0.688	0.727	0.744
	Aggression	0.769	0.810	0.770
ADHD	Restlessness	0.725	0.894	0.792
	Fidgeting	0.742	0.870	0.867
	Concentration problems	0.858	0.897	0.772

*Note*: All factor loadings are significant at the *p* < 0.001 level.

Differences in latent factors means were tested by setting the latent means to 0 in the BCS70 and freely estimating the latent means in the ALSPAC and MCS. Means for the internalising (*β* = −1.08, SE = 0.02), behavioural (*β* = −1.03, SE = 0.02), and ADHD (*β* = −0.55, SE = 0.03) factors were all significantly lower (*p* < 0.001) in the ALSPAC compared to the BCS70. In the MCS, the latent means for the internalising (*β* = 0.23, SE = 0.02) and ADHD factors (*β* = 0.60, SE = 0.03) were significantly higher (*p* < 0.001) than the BCS70, but the behavioural factor mean did not significantly differ (*β* = 0.01, SE = 0.03).

### Mental health outcomes

Factor scores from the partial scalar invariance model for the internalising, behaviour, and ADHD factors were saved. The SEP latent classes were dummy‐coded with the ‘*Prestige‐Resource Advantaged*’ class as the reference category. Then, a regression model with dummy‐coded SEP latent classes and sociodemographic covariates was specified to predict mental health factor scores.

#### BCS70

**FIGURE 1:**
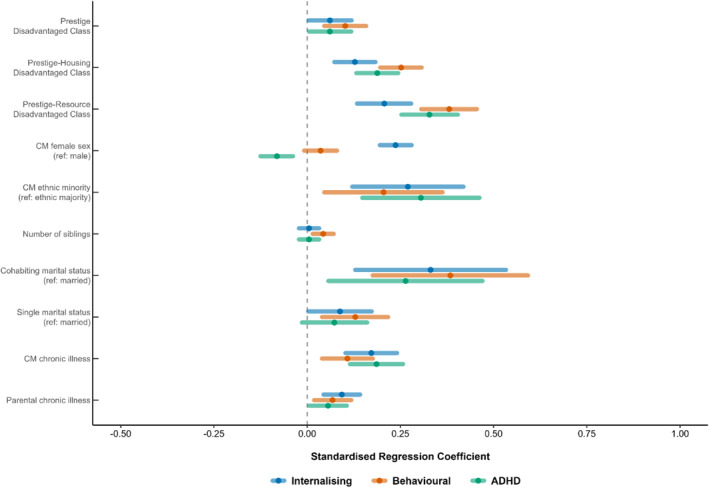
British Cohort Study standardised regression coefficient plot for mental health outcomes. SEP latent classes were dummy‐coded with the *‘Prestige‐Resource Advantaged’* class as the reference class.

In the BCS70, *‘Prestige Disadvantaged’*, *‘Prestige‐Housing Disadvantaged’*, and *‘Prestige‐Resource Disadvantaged’* class membership were all significantly related to higher levels of internalising, behavioural, and ADHD symptoms compared to the *‘Prestige‐Resource Advantaged’* class. However, for all mental health outcomes, the largest standardised regression coefficients were associated with the *‘Prestige‐Resource Disadvantaged’* class and the lowest were associated with the ‘*Prestige Disadvantaged*’ class. Additionally, female sex was strongly associated with internalising symptoms and male sex was associated with ADHD symptoms. All mental health outcomes were positively associated with CM chronic illness, parental chronic illness, ethnic minority status, and cohabiting marital status. Furthermore, single parenting was positively associated with internalising and behavioural symptoms. Number of siblings was also significantly related to behavioural symptoms. See Figure 1 for a standardised regression coefficient plot or Table S3 for a table of standardised regression coefficients.

#### ALSPAC

**FIGURE 2:**
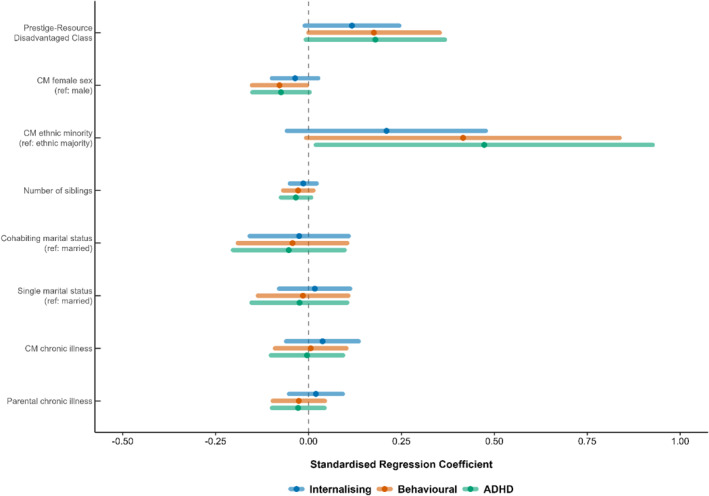
Avon Longitudinal Study of Parents and Children standardised regression coefficient plot for mental health outcomes. SEP latent classes were dummy‐coded with the *‘Prestige‐Resource Advantaged’* class as the reference class.

In the ALSPAC, few of the included predictors were significantly related to mental health symptoms. However, the *‘Prestige‐Resource Disadvantaged’* class was positively associated with behavioural symptoms. Male sex was also significantly related to behavioural symptoms and ethnic minority status was significantly related to ADHD symptoms. All other associations were non‐statistically significant. See Figure 2 for a standardised regression coefficient plot. Table S4 contains a table of standardised regression coefficients.

#### MCS

**FIGURE 3:**
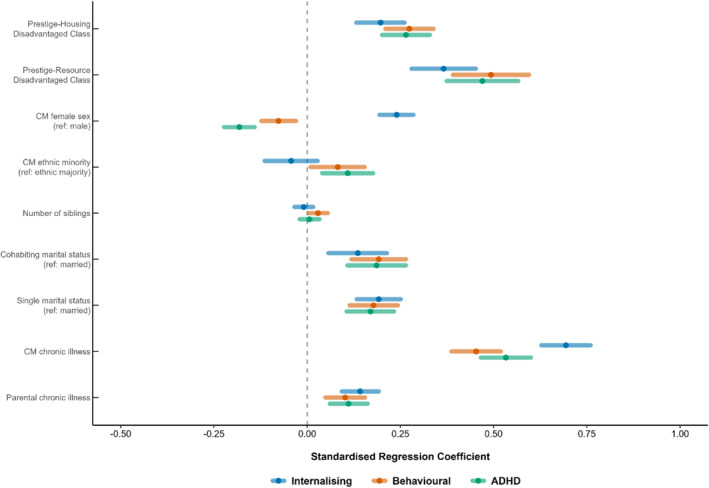
Millennium Cohort Study standardised regression coefficient plot for mental health outcomes. SEP latent classes were dummy‐coded with the *‘Prestige‐Resource Advantaged’* class as the reference class.

In the MCS, the *‘Prestige‐Housing Disadvantaged’* and *‘Prestige‐Resource Disadvantaged’* classes had higher internalising, behavioural, and ADHD symptoms compared to the *‘Prestige‐Resource Advantaged’* class. Internalising symptoms were also associated with female sex, single or cohabiting marital status, CM chronic illness, and parental chronic illness. Both behavioural and ADHD symptoms were related to male sex, ethnic minority status, single or cohabiting marital status. Behavioural symptoms were also positively associated with number of siblings. See Figure 3 for a standardised regression coefficient plot. Table S5 contains a table of standardised regression coefficients.

## DISCUSSION

This study had two primary aims: to, (1) retrospectively harmonise mental health items across three birth cohorts and test for measurement invariance, and (2) test mental health symptoms as a distal outcome of SEP latent classes from each cohort. The key findings can be summarised succinctly. Firstly, partial scalar invariance suggests that the means of the internalising, behavioural, and ADHD latent factors are comparable across cohorts (Putnick & Bornstein, 2016). Additionally, the ALSPAC cohort had the lowest factor of mental health problems for all symptom domains across all cohorts. Mean factor scores for internalising and ADHD factors were significantly higher in the MCS compared to the BCS70, indicating that the highest levels of these symptoms were observed in recent cohorts.

One explanation for this may be that greater awareness and knowledge of mental health symptoms in recent decades has led to increased reporting of symptoms, which some previous research has suggested (Oswalt et al., 2020). However, many other studies have found that the increases that have been observed are limited to specific symptoms or subgroups of the population, indicating that this is not a general result of increased awareness (Bor et al., 2014; Collishaw et al., 2010; Gore Langton et al., 2011). Indeed, in the present study, the increases were observed for internalising and ADHD symptoms, but not behavioural problems. Although prior research has suggested increases in conduct and behavioural problems between 1974 and 1999 (Collishaw et al., 2004), the present study used data from 2018 to 2019. Furthermore, Collishaw et al. (2004) conducted a calibration study to compare the Rutter Behaviour Scale and SDQ scores, yet measurement invariance was not evaluated. Since partial measurement invariance was supported in the present study, changes in symptoms observed cannot entirely be attributed to differences in how the scales were interpreted across cohorts. Altogether, the present study's findings do not suggest that the increase in mental health difficulties observed is due to increased publick awareness.

The relationship between latent class membership and mental health outcomes was also evaluated. Contrary to hypotheses, there was only partial support for a stronger relationship between internalising and externalising symptoms and the *‘Prestige‐Resource Disadvantaged’* class in each cohort. In both the BCS70 and MCS cohorts, internalising, behavioural, and ADHD symptoms were significantly related to the *‘Prestige‐Resource Disadvantaged’* class; however, in the ALSPAC, only behavioural symptoms were significant. Consistent with prior predictions, there was support for a stronger relationship between externalising symptoms (i.e., behavioural and ADHD symptoms) and the *‘Prestige‐Resource Disadvantaged’* class compared to internalising symptoms across all three cohorts. Standardised regression coefficients for behavioural and ADHD symptoms were larger than internalising symptoms in the BCS70 and MCS. As noted above, behavioural symptoms were associated with the *‘Prestige‐Resource Disadvantaged’* class in the ALSPAC, yet internalising symptoms were not significantly related.

Consistent with prior predictions, internalising and externalising symptoms appear to be more strongly related to the *‘Prestige‐Resource Disadvantaged’* class in the MCS cohort compared to the BCS70 and ALSPAC. Standardised regression coefficients for the MCS appear larger for all three symptom clusters compared to the BCS70. In the ALSPAC, only behavioural symptoms were significant with a smaller standardised regression coefficient than the other cohorts. This suggests that the relationship between the most relatively disadvantaged class and adolescent mental health difficulties may be stronger in recent years, consistent with McElroy, Tibber, et al.’s (2023) findings. The stronger relationship between socioeconomic disadvantage and mental health symptoms may partially explain the increased mental health difficulties observed in the MCS cohort. Simultaneously, the relationship between certain sociodemographic covariates, particularly single parenting and CM chronic illness was stronger in the MCS compared to the BCS70, which may also contribute to the increased mental health difficulties observed. It was not possible to directly test for differences in mental health outcomes between the cohorts using multi‐group LCA due to differences in model features (e.g., weights, stratum), so these results should be interpreted cautiously. Despite the limitations of this evidence, the results imply that although the *‘Prestige‐Resource Disadvantaged’* class in each cohort differ in size and characteristics, the adolescents from these families experience a greater burden of mental health difficulties, even more so in recent decades.

Additionally, several sociodemographic covariates emerged as consistent predictors of mental health symptoms across the BCS70 and MCS cohorts. Female sex was associated with internalising symptoms and male sex was associated with behavioural or ADHD symptoms. This is consistent with existing research suggesting a predisposition for females to develop mood disorders, whereas males are more likely to display behavioural difficulties (Boyd et al., 2015; Yoon et al., 2023). However, sex was not significantly related to internalising or ADHD symptoms in the ALSPAC, contrary to some previous findings (Armitage et al., 2023). Notably, prior studies have used the entire SDQ or all items in a single subscale whereas this study used a select number of items which were candidates for harmonisation. It is possible that SDQ items not included in this study underlie the sex differences observed in prior work. Additionally, it is possible that differences in the selection criteria and missing data strategies account for the disparate findings. Other significant covariates included single or cohabiting marital status, which was related to most mental health outcomes in both cohorts, supporting prior findings that children from non‐traditional family types are more likely to have greater emotional and behavioural difficulties (Brown, 2004; Ford et al., 2007). Ethnic minority status was also related to greater mental health difficulties. Evidence from prior research is heterogenous regarding differences between ethnic groups; some studies have found that ethnic minorities are more likely to have mental health difficulties compared to white children (Ahmad et al., 2022; Bains & Gutman, 2021), whereas others have found the opposite (A. Goodman et al., 2008; Terhaag et al., 2021). However, these differences vary by ethnic minority group (e.g., Pakistani, Black African, Mixed, etc.) and it was not possible to examine distinct ethnic minority groups in this study due to low frequencies (*n* < 20). Thusly, future research should investigate if certain ethnic minority groups are more likely to have mental health difficulties compared to others. Furthermore, CM and parental chronic illness were also significant predictors of mental health symptoms in both cohorts, which is supported by similar findings in the literature (Finning et al., 2022; Sieh et al., 2010). Although most of the associations identified in the BCS70 and MCS were not replicated in the ALSPAC, this may be at least partially attributable to the nature of the sample. The ALSPAC is known to differ from the national population with an over‐representation of affluent and white households (Boyd et al., 2013), which may underlie some of the discrepant findings.

Although this study has numerous strengths, some limitations must be considered. Firstly, parent‐reported versions of the Rutter Behaviour Scales and SDQ were compared across cohorts. Parent‐reported mental health symptoms may be prone to particular biases; for example, mothers with mental health difficulties may report greater symptom severity in their children compared to other raters (De Los Reyes & Kazdin, 2005; J. M. Müller et al., 2011). Previous research also suggests that parent‐reported SDQ scores were more strongly related to socioeconomic characteristics than child‐reported scores (Lewis et al., 2015). Although comparable self‐report versions of mental health scales were not available at similar time points across these cohorts, future research should seek to extend these findings to adolescent self‐reported mental health difficulties. Additionally, there are some variables that were not examined in this study that are known to affect mental health outcomes and are disproportionately concentrated in disadvantaged groups, including parental psychopathology, parenting practices, or other adversities (Cattan et al., 2022; Gore Langton et al., 2011; Washbrook et al., 2014), amongst others. This study did not examine the potential causal mechanisms linking socioeconomic disadvantage and adolescent mental health, but future research should test if these pathways differ between cohorts. Finally, the ALSPAC cohort sample is not nationally representative, thusly the results observed in the ALSPAC cohort cannot be extended to the rest of the UK.

In conclusion, growing up in socioeconomic disadvantage may confer greater risk of mental health difficulties for adolescents in the 2000s compared to the 1970s and 1990s. Importantly, this effect does not seem to be a result of increased publick awareness; rather the results suggest that SEP and certain sociodemographic characteristics may be more strongly linked to poor mental health in recent decades. The rising prevalence of adolescent mental health difficulties is focus of concern since it is known to impact on youth's transition to adulthood (Copeland et al., 2015) and their future health, wellbeing, and life outcomes (Mensah & Hobcraft, 2008; Thompson et al., 2023). Future research should expand this study's findings to investigate potential mediators of the relationship between socioeconomic disadvantage and adolescent mental health difficulties such as parental psychopathology, parental substance use, parenting styles and family functioning (Blume et al., 2021). Nonetheless, early life socioeconomic disadvantage is an important determinant of adolescent mental health, and both researchers and policymakers should seek ways to reduce the burden of disadvantage.

## AUTHOR CONTRIBUTIONS

**Caitlyn Rawers**: Conceptualization; investigation; writing—original draft; methodology; visualization; writing—review and editing; formal analysis. **Orla McBride**: Conceptualization; supervision; resources; writing—review and editing. **Jamie Murphy**: Conceptualization; writing—review and editing; supervision. **Eoin McElroy**: Conceptualization; methodology; supervision; resources; writing—review and editing.

## CONFLICT OF INTEREST STATEMENT

The authors declare no conflicts of interest.

## ETHICAL CONSIDERATIONS

This study involved secondary analysis of anonymised data from three cohort studies. Ethical approval for the original data collection was obtained by the BCS70 from the NHS Research Ethics Committee (Shepherd & Gilbert, 2019a), the ALSPAC from the ALSPAC Ethics and Law Committee and Local Research Ethics Committees (University of Bristol, n.d.), and the MCS from the NHS Research Ethics Committee (Shepherd & Gilbert, 2019b). Informed consent was obtained from all cohort members at the time of data collection for the respective studies. The present analysis did not require additional ethical approval, as it used fully anonymised data accessed in accordance with the data governance and access procedures of each cohort study. Specific approval dates and reference numbers are held by the original study teams and were not reissued for this secondary analysis; however, summaries are available from the references listed above.

## Supporting information

Supporting Information S1

Tables S1–S5

## Data Availability

Data from the BCS70 are available from the UK Data Service repository for free: https://doi.org/10.5255/UKDA‐Series‐200001. Data from the MCS are also available from the UK Data Service repository for free: https://doi.org/10.5255/UKDA‐Series‐2000031. Data from the ALSPAC cohort are available from the University of Bristol for a fee, following a research proposal: https://www.bristol.ac.uk/alspac/researchers/access/.
