# Supplementary material for: Investigating the toll of childhood socioeconomic disadvantage on adolescent mental health in three UK cohorts
Source: JCPP Adv. 2026 Jul 11:e70131. Online ahead of print. doi: 10.1002/jcv2.70131 (PMC13355002; doi:10.1002/jcv2.70131)
Supplement: Supplementary file 2 — Tables S1–S5 [file JCV2-9999-e70131-s001.docx]

**Investigating the toll of childhood socioeconomic disadvantage on adolescent mental health in three UK cohorts**

**Supporting Information**

**Table S1**

*Latent Class Conditional Item Probabilities for SEP Indicators in BCS70, ALSPAC, and MCS Cohorts*

| **BCS70** | **Social Class** | **Income** | **Housing Tenure** | **Education** | **Car Access** | **Mould/Damp** | **Overcrowding** |
| --- | --- | --- | --- | --- | --- | --- | --- |
| *Prestige-Resource Advantaged* | .01 | .02 | .07 | .32 | .04 | .08 | .01 |
| *Prestige Disadvantaged* | .61 | .12 | .00 | .82 | .08 | .03 | .03 |
| *Prestige-Housing Disadvantaged* | .62 | .22 | .72 | .93 | .25 | .26 | .02 |
| *Prestige-Resource Disadvantaged* | .86 | .73 | .81 | .96 | .81 | .38 | .06 |
| **ALSPAC** | **Social Class** | **Income** | **Housing Tenure** | **Education** | **Car Access** | **Mould/Damp** | **Overcrowding** |
| *Prestige-Resource Advantaged* | .11 | .05 | .03 | .16 | .02 | .36 | .00 |
| *Prestige-Resource Disadvantaged* | .35 | .87 | .67 | .41 | .14 | .34 | .01 |
| **MCS** | **Social Class** | **Income** | **Housing Tenure** | **Education** | **Car Access** | **Mould/Damp** | **Overcrowding** |
| *Prestige-Resource Advantaged* | .08 | .08 | .12 | .11 | .01 | .12 | .01 |
| *Prestige-Housing Disadvantaged* | .79 | .29 | .46 | .60 | .00 | .19 | .01 |
| *Prestige-Resource Disadvantaged* | .83 | .36 | .94 | .71 | .58 | .31 | .03 |

*Note.* Higher values reflect higher probability of disadvantage for that indicator (e.g., a value of .70 for income indicates that the latent class has a 70% probability of being low income).

**Table S2**

*OOB Errors for Partial Missing Data Imputed with missForest*

| **Variable** | **Error Type** | **OOB Error** | | |
| --- | --- | --- | --- | --- |
|  |  | **BCS70** | **ALSPAC** | **MCS** |
| **SEP Indicators** |  |  |  |  |
| *Social class* | PFC | .291 | .159 | .215 |
| *Income* | PFC | .155 | .160 | .166 |
| *Housing tenure* | PFC | .238 | .099 | .169 |
| *Educational qualifications* | PFC | .203 | .182 | .236 |
| *Car access* | PFC | .196 | .064 | .102 |
| *Mould or damp in accommodation* | PFC | .187 | .405 | .176 |
| *Overcrowding* | PFC | .028 | .004 | .014 |
| **Covariates** |  |  |  |  |
| *Marital status* | PFC | .068 | .092 | .053 |
| *Number of siblings* | RMSE | .266 | .660 | - |
| *CM chronic illness* | PFC | .000 | .157 | .143 |
| *Parental chronic illness* | PFC | .000 | .251 | .165 |
| **Mental Health Outcomes** |  |  |  |  |
| *Low mood* | PFC | .162 | .117 | .204 |
| *Worry* | PFC | .328 | .239 | .328 |
| *Fear/anxiety* | PFC | .284 | .168 | .308 |
| *Irritability/temper* | PFC | .379 | .278 | .325 |
| *Dishonesty* | PFC | .144 | .076 | .124 |
| *Disobedience* | PFC | .222 | .265 | .297 |
| *Stealing* | PFC | .076 | .258 | .034 |
| *Aggression* | PFC | .111 | .150 | .044 |
| *Restlessness* | PFC | .165 | .242 | .249 |
| *Fidgeting* | PFC | .162 | .124 | .181 |
| *Concentration problems* | PFC | .178 | .221 | .317 |

*Note.* PFC = proportion falsely classified (for categorical variables), RMSE = root mean square error (for continuous variables).

*missForest* returns the mean square error (MSE) for continuous variables which are expressed as the square of units of the imputed variable. The MSE was converted to RMSE for interpretability so RMSE errors are expressed in the unit of the imputed variable. OOB errors range from 0 to 1, values closer to 0 are desirable (Stekhoven, 2011).

There were no partial missing data for number of siblings in the MCS.

**Table S3**

*BCS70 Mental Health Outcomes Standardised Regression Coefficients*

| **Outcome** | **Covariates** | ***β*** | ***SE*** | **95% C.I.** | | ***p*** |
| --- | --- | --- | --- | --- | --- | --- |
|  |  |  |  | **LL** | **UL** |  |
| ***Internalising*** | ***Prestige Disadvantaged Class*** | .061 | .029 | .004 | .119 | .037* |
|  | ***Prestige-Housing Disadvantaged Class*** | .128 | .028 | .073 | .183 | < .001*** |
|  | ***Prestige-Resource Disadvantaged Class*** | .207 | .037 | .134 | .279 | < .001*** |
|  | *CM female sex (ref: male)* | .237 | .022 | .195 | .280 | < .001*** |
|  | *CM ethnic minority (ref: white)* | .270 | .076 | .121 | .419 | < .001*** |
|  | *Number of siblings* | .005 | .014 | -.022 | .032 | .715 |
|  | *Cohabiting marital status (ref: married)* | .331 | .103 | .129 | .533 | < .01** |
|  | *Single marital status (ref: married)* | .088 | .043 | .003 | .173 | .044* |
|  | *CM chronic illness* | .172 | .035 | .103 | .241 | < .001*** |
|  | *Parental chronic illness* | .093 | .025 | .044 | .142 | < .001*** |
| ***Behavioural*** | ***Prestige Disadvantaged Class*** | .102 | .029 | .046 | .158 | < .001*** |
|  | ***Prestige-Housing Disadvantaged Class*** | .252 | .028 | .196 | .307 | < .001*** |
|  | ***Prestige-Resource Disadvantaged Class*** | .381 | .038 | .306 | .455 | < .001*** |
|  | *CM female sex (ref: male)* | .036 | .022 | -.008 | .080 | .105 |
|  | *CM ethnic minority (ref: white)* | .205 | .081 | .046 | .363 | .011* |
|  | *Number of siblings* | .043 | .014 | .015 | .071 | < .01** |
|  | *Cohabiting marital status (ref: married)* | .384 | .106 | .176 | .592 | < .001*** |
|  | *Single marital status (ref: married)* | .129 | .045 | .040 | .217 | < .01** |
|  | *CM chronic illness* | .108 | .034 | .040 | .176 | < .01** |
|  | *Parental chronic illness* | .068 | .025 | .019 | .118 | < .01** |
| ***ADHD*** | ***Prestige Disadvantaged Class*** | .061 | .029 | .005 | .118 | .033* |
|  | ***Prestige-Housing Disadvantaged Class*** | .188 | .028 | .132 | .244 | < .001*** |
|  | ***Prestige-Resource Disadvantaged Class*** | .328 | .038 | .253 | .404 | < .001*** |
|  | *CM female sex (ref: male)* | -.081 | .022 | -.125 | -.038 | < .001*** |
|  | *CM ethnic minority (ref: white)* | .305 | .080 | .148 | .462 | < .001*** |
|  | *Number of siblings* | .005 | .014 | -.022 | .032 | .717 |
|  | *Cohabiting marital status (ref: married)* | .264 | .105 | .057 | .470 | .012* |

**Table S3 (continued)**

*BCS70 Mental Health Outcomes Standardised Regression Coefficients*

| **Outcome** | **Covariates** | ***β*** | ***SE*** | **95% C.I.** | | ***p*** |
| --- | --- | --- | --- | --- | --- | --- |
|  |  |  |  | **LL** | **UL** |  |
|  | *Single marital status (ref: married)* | .073 | .045 | -.014 | .161 | .101 |
|  | *CM chronic illness* | .186 | .036 | .115 | .257 | < .001*** |
|  | *Parental chronic illness* | .056 | .026 | .006 | .106 | .027* |

* *p* < .05, ** *p* < .01, *** *p* < .001

**Table *S*4**

*ALSPAC Mental Health Outcomes Standardised Regression Coefficients*

| **Outcome** | **Covariates** | ***β*** | ***SE*** | **95% C.I.** | | ***p*** |
| --- | --- | --- | --- | --- | --- | --- |
|  |  |  |  | **LL** | **UL** |  |
| ***Internalising*** | ***Prestige-Resource Disadvantaged Class*** | .117 | .065 | -.010 | .244 | .070 |
|  | *CM female sex (ref: male)* | -.036 | .032 | -.098 | .026 | .254 |
|  | *CM ethnic minority (ref: white)* | .210 | .136 | -.058 | .477 | .124 |
|  | *Number of siblings* | -.014 | .018 | -.050 | .022 | .458 |
|  | *Cohabiting marital status (ref: married)* | -.025 | .068 | -.158 | .108 | .715 |
|  | *Single marital status (ref: married)* | .017 | .049 | -.079 | .112 | .733 |
|  | *CM chronic illness* | .038 | .050 | -.060 | .135 | .447 |
|  | *Parental chronic illness* | .020 | .037 | -.052 | .092 | .590 |
| ***Behavioural*** | ***Prestige-Resource Disadvantaged Class*** | .176 | .090 | .000 | .353 | .050* |
|  | *CM female sex (ref: male)* | -.078 | .038 | -.152 | -.004 | .040* |
|  | *CM ethnic minority (ref: white)* | .416 | .215 | -.006 | .837 | .053 |
|  | *Number of siblings* | -.028 | .021 | -.068 | .013 | .180 |
|  | *Cohabiting marital status (ref: married)* | -.043 | .075 | -.190 | .104 | .568 |
|  | *Single marital status (ref: married)* | -.015 | .062 | -.136 | .107 | .815 |
|  | *CM chronic illness* | .006 | .049 | -.090 | .102 | .904 |
|  | *Parental chronic illness* | -.026 | .036 | -.096 | .044 | .468 |
| ***ADHD*** | ***Prestige-Resource Disadvantaged Class*** | .180 | .095 | -.007 | .367 | .060 |
|  | *CM female sex (ref: male)* | -.074 | .039 | -.151 | .003 | .060 |
|  | *CM ethnic minority (ref: white)* | .473 | .231 | .020 | .926 | .041* |
|  | *Number of siblings* | -.034 | .021 | -.074 | .007 | .101 |
|  | *Cohabiting marital status (ref: married)* | -.053 | .076 | -.203 | .097 | .488 |
|  | *Single marital status (ref: married)* | -.024 | .065 | -.153 | .104 | .708 |
|  | *CM chronic illness* | -.004 | .049 | -.101 | .093 | .932 |
|  | *Parental chronic illness* | -.028 | .036 | -.098 | .043 | .441 |

* *p* < .05

**Table S5**

*MCS Mental Health Outcomes Standardised Regression Coefficients*

| **Outcome** | **Covariates** | ***β*** | ***SE*** | | **95% C.I.** | | ***p*** |
| --- | --- | --- | --- | --- | --- | --- | --- |
|  |  |  |  |  | **LL** | **UL** |  |
| ***Internalising*** | ***Prestige-Housing Disadvantaged Class*** | .197 | | .033 | .132 | .261 | < .001*** |
|  | ***Prestige-Resource Disadvantaged Class*** | .366 | | .044 | .280 | .452 | < .001*** |
|  | *CM female sex (ref: male)* | .240 | | .023 | .194 | .285 | < .001*** |
|  | *CM ethnic minority (ref: white)* | -.043 | | .036 | -.114 | .028 | .234 |
|  | *Number of siblings* | -.009 | | .013 | -.034 | .016 | .492 |
|  | *Cohabiting marital status (ref: married)* | .136 | | .040 | .057 | .214 | < .01** |
|  | *Single marital status (ref: married)* | .192 | | .030 | .133 | .251 | < .001*** |
|  | *CM chronic illness* | .694 | | .034 | .628 | .760 | < .001*** |
|  | *Parental chronic illness* | .142 | | .025 | .093 | .192 | < .001*** |
| ***Behavioural*** | ***Prestige-Housing Disadvantaged Class*** | .274 | | .033 | .210 | .339 | < .001*** |
|  | ***Prestige-Resource Disadvantaged Class*** | .493 | | .052 | .391 | .595 | < .001*** |
|  | *CM female sex (ref: male)* | -.077 | | .024 | -.123 | -.030 | < .01** |
|  | *CM ethnic minority (ref: white)* | .082 | | .037 | .009 | .154 | .028* |
|  | *Number of siblings* | .029 | | .013 | .004 | .055 | < .001*** |
|  | *Cohabiting marital status (ref: married)* | .192 | | .037 | .119 | .265 | < .001*** |
|  | *Single marital status (ref: married)* | .178 | | .033 | .114 | .243 | < .001*** |
|  | *CM chronic illness* | .453 | | .034 | .387 | .519 | < .001*** |
|  | *Parental chronic illness* | .102 | | .027 | .049 | .155 | < .001*** |
| ***ADHD*** | ***Prestige-Housing Disadvantaged Class*** | .265 | | .033 | .201 | .329 | < .001*** |
|  | ***Prestige-Resource Disadvantaged Class*** | .470 | | .049 | .374 | .566 | < .001*** |
|  | *CM female sex (ref: male)* | -.182 | | .021 | -.223 | -.141 | < .001*** |
|  | *CM ethnic minority (ref: white)* | .109 | | .035 | .040 | .177 | < .01** |
|  | *Number of siblings* | .006 | | .014 | -.020 | .033 | .644 |
|  | *Cohabiting marital status (ref: married)* | .186 | | .040 | .108 | .265 | < .001*** |
|  | *Single marital status (ref: married)* | .170 | | .032 | .106 | .233 | < .001*** |

**Table S5 (continued)**

*MCS Mental Health Outcomes Standardised Regression Coefficients*

| **Outcome** | **Covariates** | ***β*** | ***SE*** | **95% C.I.** | | ***p*** |
| --- | --- | --- | --- | --- | --- | --- |
|  |  |  |  | **LL** | **UL** |  |
|  | *CM chronic illness* | .533 | .034 | .466 | .600 | < .001*** |
|  | *Parental chronic illness* | .111 | .026 | .061 | .162 | < .001*** |

* *p* < .05, ** *p* < .01, *** *p* < .001
